# Simple versus composite indicators of socioeconomic status in resource allocation formulae: the case of the district resource allocation formula in Malawi

**DOI:** 10.1186/1472-6963-10-6

**Published:** 2010-01-06

**Authors:** Gerald Manthalu, Dominic Nkhoma, Sanderson Kuyeli

**Affiliations:** 1 Department of Planning and Policy Development, Ministry of Health, P.O Box 30377, Lilongwe 3, Malawi

## Abstract

**Background:**

The district resource allocation formula in Malawi was recently reviewed to include stunting as a proxy measure of socioeconomic status. In many countries where the concept of need has been incorporated in resource allocation, composite indicators of socioeconomic status have been used. In the Malawi case, it is important to ascertain whether there are differences between using single variable or composite indicators of socioeconomic status in allocations made to districts, holding all other factors in the resource allocation formula constant.

**Methods:**

Principal components analysis was used to calculate asset indices for all districts from variables that capture living standards using data from the Malawi Multiple Indicator Cluster Survey 2006. These were normalized and used to weight district populations. District proportions of national population weighted by both the simple and composite indicators were then calculated for all districts and compared. District allocations were also calculated using the two approaches and compared.

**Results:**

The two types of indicators are highly correlated, with a spearman rank correlation coefficient of 0.97 at the 1% level of significance. For 21 out of the 26 districts included in the study, proportions of national population weighted by the simple indicator are higher by an average of 0.6 percentage points. For the remaining 5 districts, district proportions of national population weighted by the composite indicator are higher by an average of 2 percentage points. Though the average percentage point differences are low and the actual allocations using both approaches highly correlated (*ρ *of 0.96), differences in actual allocations exceed 10% for 8 districts and have an average of 4.2% for the remaining 17. For 21 districts allocations based on the single variable indicator are higher.

**Conclusions:**

Variations in district allocations made using either the simple or composite indicators of socioeconomic status are not statistically different to recommend one over the other. However, the single variable indicator is favourable for its ease of computation.

## Background

Health care systems adopt various ways of allocating resources to sub-national areas and agencies. The four commonly used methods are: i) political patronage ii) historical allocations iii) bids by local governments and iv) needs-based resource allocation formulae [1]. Political patronage entails government rewarding loyal constituencies and potential strongholds. Under historical allocation, funds are allocated according to past year's expenditures adjusted by inflation and efficiency changes. Under the third mechanism, central Government will allocate resources by scrutinizing uncapped budget submissions made by Local Authorities, funding activities that are in line with overarching national strategies. The needs based resource allocation formula entails central Government allocating resources based on a mathematical formula that integrates need for health care. The allocation of resources usually takes place in the context of devolution where services are delivered by lower level jurisdictions.

Formula funding has become a preferred method of allocating resources in many publicly financed health systems. Some of the merits of needs-based resource allocation formulae include that resources are allocated to areas of high priority, where they can secure the highest marginal health benefit. Besides, a well designed formula allows financial resources to be allocated to providers in proportion to services that they deliver. Formula funding also presents a widely accepted mechanism for setting budgets for devolved organizations [1].

In most African countries, health expenditures across different districts or regions have not matched with need for health care [2]. This is largely attributable to inheritance of past inequitable systems and allocation patterns that ensued from such systems. Explicit resource allocation formulae hence ensure that the share of total resources allocated to an area is based on indicators of relative need for health care [2].

The resource allocation formula for funding districts in Malawi was reviewed in 2007/08 with a view to improving its ability to reflect need and capture differential costs of service provision across districts. The basic indicator of health care need in the formula is population by district which is weighted by stunting. Stunting was selected as an indicator of overall socioeconomic status of a district such that districts with higher rates have lower socioeconomic status overall and vice versa. It is referred to as a simple or single variable indicator in this paper. It is expressed as percentage of children under-five, by district, whose height-for-age falls below minus 3 standard deviations from the median height-for-age of a standard reference population used in the Multiple Indicator Cluster Survey 2006. Stunting has been recommended as a reliable measure of overall socioeconomic deprivation [3,4].

It has been argued that a single variable indicator of need can be susceptible to rapid changes or fluctuations which might not reflect actual patterns of need [5]. Its advantage, however, is that it is much easier to compute. The composite indicator, on the other hand, requires differential weighting of variables which can be achieved by either employing expert opinion or through a principal components analysis. In many countries where the concept of need has been incorporated in resource allocation, composite indicators of socioeconomic status such as deprivation and asset indices have been used (see Zere et al. 2007, McIntyre et al. 2000). This paper examines, using the Malawi district resource allocation formula, whether there are differences between weighting population with simple and composite indicators of socioeconomic status in terms of allocations made to districts, holding all other factors in the formula constant.

### Brief country profile

Malawi is a sub-Saharan country that covers an area of 118, 484 square kilometres. It has 28 administrative districts, of which 4 are cities. The 2008 population and housing census estimated total population at 13,066,320 [6]. Life expectancy is low and is projected at 48 years for both males and females [7]. Table 1 presents some of the health and development indicators for the country [6-12]. GDP per capita for the year 2008 was estimated at US$ 850 purchasing power parity (PPP). However, the median per capita income of the richest income decile (i.e. the richest 10%) is about eight times that of the poorest decile. According to the 2008 Welfare Monitoring Survey, 40% of Malawians are classed as poor. As in many developing countries, percapita health expenditure is low. It was estimated at US$25 by the Malawi National Health Accounts (2008) far below the World Health Organization (WHO) recommended minimum of US$34 per capita.

**Table 1:** Health and development indicators for Malawi

Life expectancy at birth, male/female (years)	46.9/49.5
Total fertility rate	6.3
One year olds fully immunized against measles, 2007/08 (%)	85
Infant mortality rate (per 1000 live births), 2006 (%)	69
Under-five mortality rate (per 1000 live births), 2006	118
Maternal mortality ratio (per 100, 000 live births)	807
Literacy rates, male/female (%)	79/59
Stunting in under-five children, 2006 (%)	46
Gross domestic product per capita, US$ PPP	850
Gini coefficient, 2005	0.38
Human development index, 2005	0.437
Adult HIV prevalence rate (15-49 years) (%)	12
Physician per population ratio	1:53,176
Nurse per population ratio	1:2,964

The epidemiological profile of Malawi is characterized by a high prevalence of communicable diseases including malaria, tuberculosis and HIV and AIDS; an increasing burden of non-communicable diseases such as cancer, hypertension, diabetes, cardiovascular diseases and mental illnesses and high incidence of maternal and child health problems.

### District resource allocation in Malawi

The district health system in Malawi is organised into two levels of care: i) primary - provided by health centres, dispensaries and community health workers and ii) secondary - provided by district hospitals and mission hospitals of equivalent capacity. Four districts have referral hospitals which provide tertiary health care but these are financed and managed separately from the district health system.

There are two categories of resources that are allocated to districts: recurrent and development. Recurrent allocations meet the operational costs of all health facilities in a district, while development funding is meant for capital expenditures such as construction or rehabilitation of health centres and purchase of big medical equipment. The district resource allocation formula applies only to the recurrent budget.

Until 2000/01, the allocation of district health funding was determined purely based on population. As such, allocations were directly proportional to district populations. The formula was subsequently revised by including the variables, poverty and under-five mortality to strengthen its ability to reflect need. In 2007/08, the formula was revisited again by replacing the previous proxy measures of need with stunting and including a variable that captures differences in the cost of delivering health care across districts. There are four variables in the current resource allocation formula and they are: stunting, index for differential costs of service provision, bed capacity and OPD utilization.

Given , the total recurrent budget for districts as determined by the Ministry of Finance, the current allocation formula is expressed as:

Where *x*_*i *_is the allocation for district *i*,

*V*_*ni *_is the value of variable n for district *i*,

*a*_*n *_is the weight for variable *V*_*n*_

The weights *a*_*i *_for the variables are currently determined by policy makers.

## Methods

### Developing a composite indicator of need

Population size in a geographical area is the primary indicator of need [13]. It can be weighted by other indicators of relative need for health such as deprivation and asset indices as well as variables that proxy burden of disease to capture other dimensions of need that cannot be captured by population only. In this study we focus on asset indices as indicators of health care need. The premise for including the asset index as an indicator of health care need is that it is a measure of socioeconomic status and there is a well established relationship between health and socioeconomic status [14]. The report of the Commission on Social Determinants of Health 2008 points out that the relationship is graded such that poorer individuals have poorer health [15].

Asset indices have been widely used because of the challenges in using standard measures of socioeconomic status that use income [16]. Income measures are difficult because they demand collecting accurate data which is expensive especially for low income countries. In addition, adjusting data for individuals who have multiple sources of income is rigorous. Further, income data does not capture income in kind e.g. maize or animals which may be traded and this leads to inaccurate estimation of actual income. Consumption expenditures are sometimes employed but these too are costly to collect [16].

Using an asset based indicator of need has got its own problems. Such measures are more reflective of longer run household socioeconomic status failing to take into account short term shocks to the household. The quality of assets is not captured and certain variables may have different relationship with socioeconomic status across the population [17].

There are important assumptions which are made when deriving the asset index. The first assumption is that variables are additive i.e. if an individual ranks poorly with respect to two or more variables, then that individual should be more deprived than one who ranks poorly on the basis of only one of them. Secondly, the variables should be weighted differently. This shows the relative importance of the variables included in the analysis [13].

A multivariate statistical technique called principal components analysis (PCA) is used to derive the asset index. Principal components analysis describes the variation of a set of variables as a set of linear combinations of the original variables, in which each consecutive linear combination is derived so as to explain as much as possible of the variation in the original data, while being uncorrelated with other linear combinations [17]. PCA works best when variables are highly correlated and their distribution varies across households [13].

Given *k *variables;

Where *PC*_*i *_is principal component *i;*

*a*_*ik *_represents the weight for the *k*^*th *^variable for the *i*^*th *^principal component

The first principal component, *PC*_1 _explains the largest possible amount of variation in the original data, subject to the constraint:

i.e. the sum of the squared weights is equal to one [17]. Typically, the asset index is assumed to be the first principal component--that is, the first linear combination.

The asset index, *A*_*i*_, for individual *i *is defined as follows:

where *a*_*ik *_is the value of asset *k *for household *i*, *a*_*k *_is the sample mean, *s*_*k *_is the sample standard deviation, and *f*_*k *_are the factor scores or weights associated with the first principal component.

In this study we developed asset indices using principal components analysis. Data were obtained from the Malawi Multiple indicator Cluster Survey (MICS) 2006. They were available for only 26 out of the 28 districts because the two districts have only been recently established. The survey treated these two districts as part of the districts to which they originally belonged before they were demarcated. Variables were expressed as categorical variables with the dichotomous responses of 'Yes', coded 1, if the household possessed that variable and 'No', coded 0, if they did not. Most of the variables that were included in the asset index have been used in similar studies in developing countries (see Houweling et. al 2003, Vyas and Kumaranayake 2006, and Zere et. al. 2007). The spearman rank correlation test was used to check correlation of the all variables included in the PCA. Variables which were not significant at the 1% significance level were excluded from the analysis. Stata/SE 10.0 and Microsoft Excel 2003 were used for the analysis.

## Results

Table 2 shows the means, standard deviations and factor scores derived from the PCA. Variables which have positive factor scores are associated with high socioeconomic status whilst the converse is true for those with negative values. The results in Table 2 show that households that use other sources of water other than piped are more likely to have low socioeconomic status, all other factors constant. Among types of toilet facility, pit latrine with no slab, composting toilet, hanging latrine and no toilet are associated with low socioeconomic status. Having a sand floor and having a thatched roof are also associated with low socioeconomic status. Ownership of a bicycle has an interesting result. It has a negative weight, however, which implies that, *ceteris paribus*, a household that owns a bicycle will be ranked lower than the one which does not. Vyas and Kumaranayake (2006) argue that this may be a result of high correlation between ownership of a bicycle and variables that are more likely to be associated with low socioeconomic status.

**Table 2:** Scoring weights derived from Principal Component Analysis

Variable Description	Mean	**Std. Dev**.	Factor Score
Car/Truck	0.009	0.093	0.068
Radio	0.934	0.248	0.016
Cell phone	0.060	0.238	0.111
Television	0.073	0.260	0.093
Bicycle	0.425	0.494	-0.007
Refrigerator	0.018	0.132	0.099
Cattle	0.476	0.499	0.040
Goat	0.654	0.476	0.024
**Source of Water Supply**			
Piped into dwelling, household uses bottled water	0.014	0.119	0.084
Piped outside dwelling	0.033	0.179	0.071
Public tap/standpipe	0.126	0.331	0.042
Tube well/borehole, tube well with powered pump, cart with small tank/drum	0.516	0.500	-0.052
Unprotected well/spring	0.170	0.376	-0.032
Surface water, rainwater collection	0.067	0.250	-0.016
**Type of toilet facility**			
Flush toilet of any type	0.020	0.140	0.092
Pit latrine with slab, pit latrine with slab and foot rest	0.101	0.301	0.037
Pit latrine without slab/open pit	0.680	0.466	-0.055
Pit latrine with slab and cover, pit latrine with slab/cover and foot rest	0.036	0.187	0.017
No toilet, composting toilet, hanging toilet/latrine	0.138	0.344	-0.025
**Type of floor material**			
Tiles, cement, carpet, wood planks, other	0.167	0.373	0.133
Sand	0.789	0.408	-0.128
**Type of roofing material**			
Thatch, sod, rustic mat, palm/bamboo	0.735	0.441	-0.124
Metal	0.229	0.420	0.126
**Source of fuel for cooking**			
Electricity, all types of gas, kerosene	0.009	0.096	0.080
Charcoal, coal	0.047	0.213	0.094
Wood	0.903	0.296	-0.116

To calculate district asset indices, individual asset indices were aggregated. Districts with negative indices are less impoverished, overall, than those with positive values. In order to incorporate the asset index in a resource allocation formula, there is need to normalize the indices. We added a value of 1.4669 which brought the least disadvantaged district, Ntchisi, to a value of 1 and the other districts to greater positive values. District populations were then multiplied by the normalized indices such that populations for highly deprived districts were considerably inflated and those for less deprived districts either remained constant or increased only slightly. Table 3 depicts the asset indices and district proportions of the national population weighted by either the asset indices or stunting rates.

**Table 3:** Simple and composite socioeconomic indicators by district

District	2008 Population	Normalised asset indices	Population weighted by asset indices	Population weighted by Stunting	Proportion of population weighted by asset indices (%)	Proportion of population weighted by stunting (%)
Balaka	316,748	1.194	378,083	361,093	1.94	2.31
Blantyre	999,491	2.616	2,614,947	1,162,408	13.41	7.44
Chikwawa	438,895	1.416	621,280	513,946	3.19	3.29
Chiradzulu	290,946	1.328	386,520	341,862	1.98	2.19
Chitipa	179,072	1.101	197,101	204,858	1.01	1.31
Dedza	623,789	1.021	636,917	799,074	3.27	5.11
Dowa	556,678	1.242	691,347	668,014	3.55	4.27
Karonga	272,789	1.479	403,586	304,978	2.07	1.95
Kasungu	616,085	1.210	745,753	732,525	3.83	4.69
Lilongwe	1,897,167	1.996	3,787,212	2,325,927	19.43	14.88
Machinga	488,996	1.095	535,673	628,849	2.75	4.02
Mangochi	803,602	1.255	1,008,684	963,519	5.17	6.16
Mchinji	456,558	1.199	547,441	594,895	2.81	3.81
Mulanje	525,429	1.447	760,077	634,718	3.90	4.06
Mwanza	94,476	1.240	117,167	109,970	0.60	0.70
Mzimba	853,305	1.731	1,477,107	1,013,726	7.58	6.49
Nkhata Bay	213,779	1.371	293,108	247,129	1.50	1.58
Nkhotakota	301,868	1.413	426,476	365,864	2.19	2.34
Nsanje	238,089	1.235	294,010	269,993	1.51	1.73
Ntcheu	474,464	1.115	529,115	581,693	2.71	3.72
Ntchisi	224,098	1.000	224,098	289,086	1.15	1.85
Phalombe	313,227	1.111	347,884	386,209	1.78	2.47
Rumphi	169,112	1.432	242,232	189,067	1.24	1.21
Salima	340,327	1.518	516,700	385,250	2.65	2.46
Thyolo	587,455	1.329	780,533	721,395	4.00	4.61
Zomba	670,533	1.391	932,631	835,484	4.78	5.34

### Comparison of results from formulae employing either the simple or composite indicators of socioeconomic status

#### i) District proportions of national population

**Figure 1:**
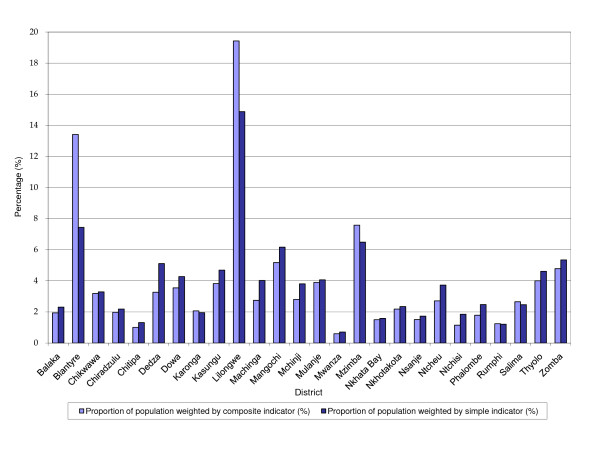
**District proportions of national population weighted by simple and composite indicators of socioeconomic status**.

Generally, district proportions of national population are lower when the composite indicator is used than the simple one. Out of the 26 districts included in the study, district proportions of national population weighted by stunting are higher for 21 districts and lower for 5 districts namely; Blantyre, Karonga, Lilongwe, Mzimba and Rumphi. For the 21 districts, mean mark up in percentage points is 0.6. For the 5 districts the mean gain in percentage points is 2. However, Blantyre and Lilongwe districts have significant gains, 5.98 and 4.55 percentage points respectively. The spearman rank correlation test for district proportions of national population weighted by the composite and simple indicators produces a *ρ *of 0.97, significant at the 1% significance level. Figure 1 depicts the district proportions of national population weighted by either the simple or composite indicators.

#### ii) Allocations

**Figure 2:**
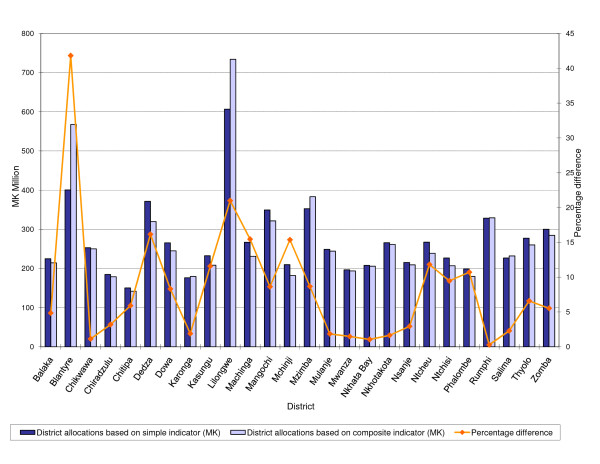
**District allocations made using formulae that employ either simple or composite indicators of socioeconomic status**.

District allocations made using formulae that contain the single variable and composite indices are depicted in figure 2. The three dimensional graph shows on the left vertical axis allocations in Malawi kwacha made using the two approaches and on the right vertical axis differences in the allocations expressed in percentage terms. For 21 districts, allocations made using the formula that employs the single variable indicator are higher. Percentage differences in actual allocations exceed 10% for 8 districts namely; Blantyre, Dedza, Kasungu, Lilongwe, Machinga, Mchinji, Ntcheu and Phalombe and have an average of 4.2% for the remaining 17. A spearman rank correlation test of the allocations made using the two approaches shows high correlation, a *ρ *of 0.96 significant at the 1% significance level.

## Discussion

The objective of this study was to assess whether there are differences in the resources allocated to districts in Malawi when either simple or composite indicators of socioeconomic status are used to weight population, holding all other factors in the allocation formula constant.

We used PCA to derive asset indices for 26 districts. The results indicate that the two types of indicators are highly correlated and so are the district allocations made using either of them. An interesting result, however, is that allocations are higher for 21 districts when the single variable indicator is used. For Blantyre, Lilongwe and Mzimba districts the values of the asset indices are considerably higher than those of the single variable indicator. A possible explanation for this could be that stunting prevalence rates for these districts were underestimated. Blantyre and Lilongwe are the country's largest cities while Mzimba district contains the third largest city, Mzuzu. These three districts have the largest populations with an average of 1,249,988 while the rest of the districts have an average of 372,654. In addition, populations in the three cities are likely to be more heterogeneous than in the rest of the districts. In the Malawi Multiple Indicator Cluster Survey 2006, district sample sizes were equal, at 1,200 households per district. For Blantyre, Lilongwe and Mzimba districts, therefore, there is possibility that, although the uniform sample sizes were calculated to provide statistically reliable estimates, they were not representative enough to effectively estimate the true population stunting rates.

## Conclusions

From the study findings, district allocations made using the simple and composite indicators of socioeconomic status are not statistically different, holding all other factors in the allocation formula constant. However, the simple indicator is advantageous because it is easy to apply and does not involve complex statistical techniques as compared to the composite indicator. The PCA technique, used in the derivation of the composite indicators, has weaknesses in that selection of variables is based on the judgement of the analyst and there is no theory that guides how weights for the variables are generated [18].

## Competing interests

The authors declare that they have no competing interests.

## Authors' contributions

GM conceived and designed the study, conducted data analysis and drafted the manuscript. DM participated in the design and analysis and critically reviewed the manuscript. SK critically reviewed the manuscript. All authors read and approved the final manuscript.

## Pre-publication history

The pre-publication history for this paper can be accessed here:

http://www.biomedcentral.com/1472-6963/10/6/prepub
